# Axillary response and diagnostic accuracy of imaging modalities after neoadjuvant chemotherapy for breast cancer (retrospective single center study)

**DOI:** 10.3389/fsurg.2026.1763488

**Published:** 2026-03-13

**Authors:** Emine Özlem Gür, Muhammet Mustafa Şafak, Melek Gökova, Merve Gürsoy, Betül Küçükzeybek, Yeliz Yılmaz Bozok, Murat Kemal Atahan

**Affiliations:** 1 Atatürk Training and Research Hospital, Department of General Surgery, Katip Çelebi University, İzmir, Türkiye; 2 Atatürk Training and Research Hospital, Department of Radiology, Katip Çelebi University, İzmir, Türkiye; 3 Atatürk Training and Research Hospital, Department of Pathology, Katip Çelebi University, İzmir, Türkiye

**Keywords:** breast cancer, imaging modalities, neoadjuvant chemotherapy, pathological response, sentinel lymph node biopsy

## Abstract

**Introduction:**

The optimal axillary approach after neoadjuvant chemotherapy (NACT) in patients with breast cancer (BC) remains controversial. In our study, we aimed to evaluate factors affecting axillary pathological complete response (pCR) in patients with biopsy-proven node-positive BC before NACT and to assess the diagnostic performance of imaging modalities in detecting residual axillary metastasis after NACT.

**Methods:**

Our sample included 142 patients with T1–3, N1–2, M0 BC who were cytologically confirmed to have axillary metastasis by ultrasonography (USG), mammography (MG), magnetic resonance imaging (MRI), and 18F-FDG positron emission tomography–computed tomography (PET-CT) and who received NACT between 2020 and 2025. Patients showing clinical or radiologic complete response in the axilla underwent sentinel lymph node biopsy (SLNB). SLNB-positive patients subsequently underwent level I–II axillary dissection (AD). Pathological, molecular, and imaging findings of the patients were analyzed.

**Results:**

After NACT, 78 patients (54.9%) had no residual axillary metastasis. HER2 positivity and progesterone receptor (PR) negativity were significantly associated with axillary pCR (p < 0.05). Luminal A and B tumors demonstrated lower response rates to NACT, whereas HER2-rich and triple-negative subtypes showed higher axillary pCR rates (85.7% and 71.4%, respectively). Among imaging modalities, the specificity values were 91.2% and 84.6% for PET-CT and USG, respectively. Negative predictive values (NPV) were 74.02% and 76.52% for PET-CT and USG, respectively.

**Discussion and conclusion:**

In PR-positive and HER2-negative tumors, the likelihood of axillary pCR is lower, and careful evaluation is warranted. USG and PET-CT may serve as a good guide for axillary lymph node assessment after NACT in patients with BC. However, given their modest sensitivity, imaging modalities should be considered complementary tools rather than substitutes for pathological axillary staging. Further prospective randomized trials are needed to define patient groups who may safely avoid axillary dissection after NACT.

## 1 Introduction

Axillary lymph node status is an important indicator to evaluate the prognosis and treatment procedures in patients with breast cancer (BC). In the surgical management of the axillary region in patients with BC, sentinel lymph node biopsy (SLNB) has become the gold standard approach in the last 15 years (1) and marked a milestone in the development of such surgery (2). SLNB is performed using blue dye and/or radiotracer in patients with clinically node-negative BC. The ACOSOG Z0011 trial showed that axillary dissection (AD) can be safely avoided in patients without any metastatic sentinel lymph nodes (SLNs) or those who have no more than two metastatic SLNs and undergo surgery as a first-line therapy (3).

Neoadjuvant chemotherapy (NACT) is commonly administered as a first-line therapy in certain subtypes of BC, especially in triple-negative and human epidermal growth factor receptor 2 (HER2)-rich tumors. When NACT was first implemented, the chief goal was to increase segmental mastectomy rates and SLNB application rates in axillary surgery (4). The response of the tumor and axilla after NACT application also provided the opportunity to evaluate the tumor's sensitivity to chemotherapy. Recently, studies have shown that patients who attain a pathological complete response (pCR) after NACT have significantly longer overall survival and disease-free survival, particularly those with triple-negative and HER2-positive BC (5).

Imaging techniques such as ultrasonography (USG), mammography (MG), magnetic resonance imaging (MRI), and 18F-fluorodeoxyglucose positron emission tomography-computed tomography (PET-CT) are used to assess axillary lymph node status after NACT. Several studies have demonstrated that the axillary response rate to NACT can be predicted with more than 60% success using USG and MRI (6, 7). SLNB can be performed in patients without any pathological lymph nodes after NACT, both clinically and radiologically. However, appropriate patient selection and the optimal technique for performing SLNB after NACT remain subjects of debate. Prospective randomized trials have demonstrated that false-negative rates are acceptable if SLNB is performed using a dual-tracer technique and excising at least three SLNs. According to current evidence, level I–II AD should be performed in patients with metastatic SLNs (8).

Targeted AD (TAD) has been introduced to further reduce false-negative rates and ensure the accurate removal of true SLNs after NACT. In this technique, a clip is placed into a cytologically confirmed metastatic lymph node before NACT, and the clipped node, along with the SLNs, is excised after the completion of chemotherapy (9). Although some studies have shown that TAD lowers false-negative rates compared with conventional SLNB (10), others have reported no significant difference between TAD and dual-tracer SLNB (9, 11). TAD has several limitations. The clip placed before NACT may migrate during treatment, and after treatment, the clip may not be visualized in some patients (12).

The aim of our study was to evaluate the tumor factors affecting the axillary response to NACT in patients with BC and biopsy-proven metastatic lymph nodes, and to assess the diagnostic accuracy of imaging modalities in detecting residual axillary metastasis following NACT. In addition, we aimed to clearly describe the patient selection process and surgical decision-making pathway after NACT to enhance methodological transparency.

## 2 Materials and methods

Between 2020 and 2025, 552 patients diagnosed with BC (i.e., clinical stages T1–3, N0–2, and M0) at our institution were retrospectively reviewed. NACT was performed as a treatment modality based on multidisciplinary tumor board decisions and international treatment guidelines in 228 patients. Of these, 167 patients had cytologically confirmed axillary metastasis using fine-needle aspiration biopsy prior to NACT and were considered eligible for further evaluation.

**Figure 1:**
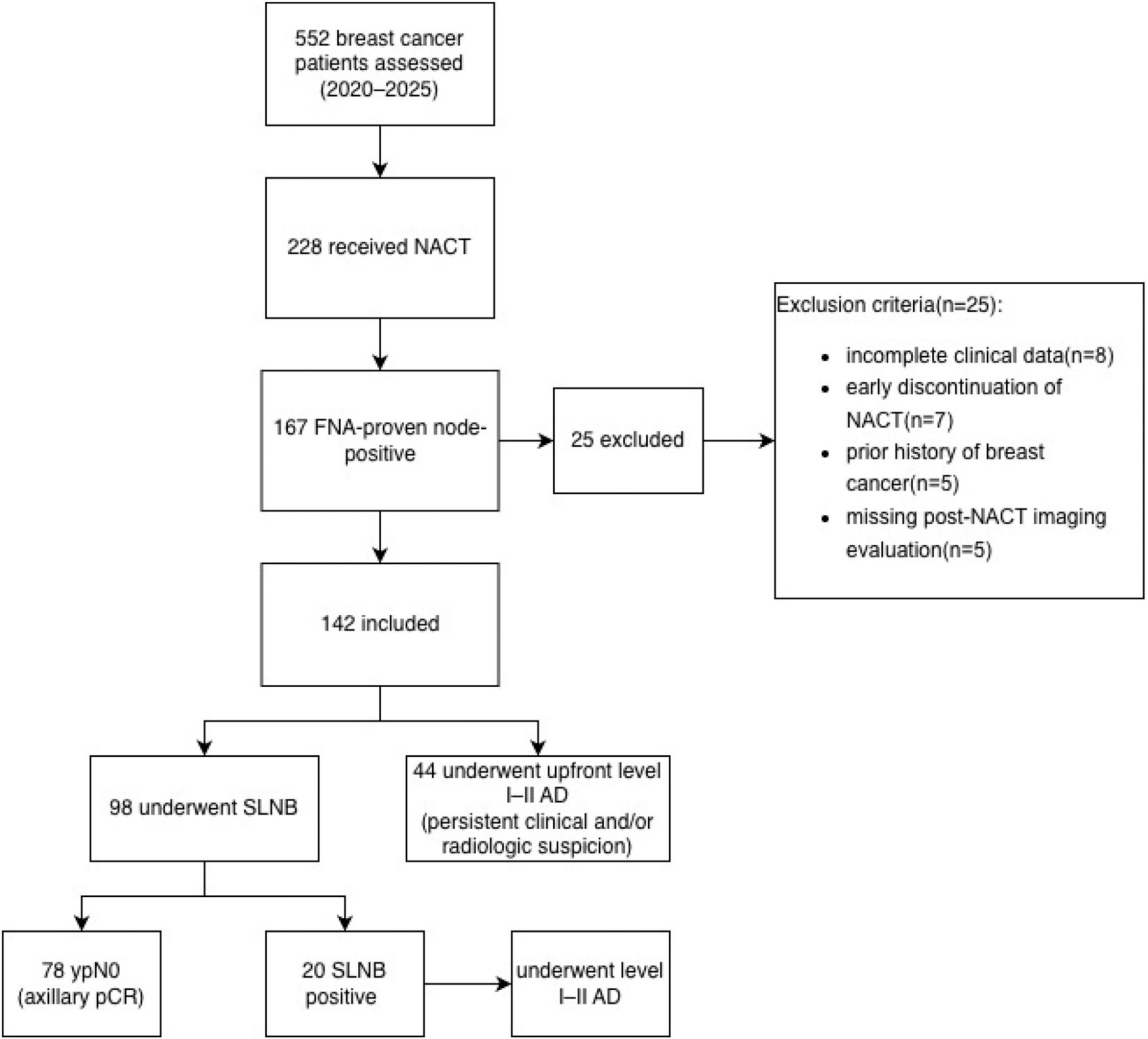
Patient selection and study flow diagram. BC, breast cancer; NACT, neoadjuvant chemotherapy; SLNB, sentinel lymph node biopsy; AD, axillary dissection.

Patients received anthracycline- and/or taxane-based chemotherapy regimens; those with HER2-positive disease also received anti-HER2 targeted therapy (i.e., trastuzumab ± pertuzumab). During the study period, immunotherapy was not routinely administered for patients with triple-negative breast cancer. HER2 positivity was determined using immunohistochemistry or fluorescence *in situ* hybridization (FISH). Patients who discontinued NACT early, had a history of treatment for BC, presented with recurrent disease, or had incomplete post-NACT data were excluded. Ultimately, 142 patients who met all inclusion criteria were enrolled in the study. Twenty-five patients were excluded due to incomplete clinical data, early discontinuation of NACT, prior history of breast cancer, or missing post-NACT imaging evaluation. The detailed patient selection process is illustrated in a CONSORT-style flow diagram (Figure 1).

All patients underwent pre-NACT staging with USG, MG, MRI, and PET-CT. Although PET-CT is not universally recommended as a standard modality for axillary staging before or after NACT in all international guidelines, it is incorporated into staging and response evaluation at our institution according to our multidisciplinary tumor board protocol. The treatment response of the primary tumor and axillary lymph nodes to NACT was evaluated using the same imaging modalities after the completion of NACT.

Imaging interpretation was performed by experienced breast radiologists according to routine institutional practice. Radiologists had access to clinical and pathological information through the hospital information system as part of the standard clinical workflow. Imaging assessment was not performed under blinded research conditions, and interobserver variability was not assessed. Radiological complete axillary response was defined as the absence of suspicious lymph node features. On USG, suspicious findings included a cortical thickness >3 mm, loss of fatty hilum, and a round morphology. On PET-CT, pathological uptake was defined as focal FDG uptake exceeding background activity.

### 2.1 Surgical procedure

Surgical management consisted of either segmental (i.e., breast-conserving) mastectomy or total mastectomy following international guidelines. Patients who demonstrated a complete clinical or radiological response in the axilla underwent SLNB using the dual-tracer technique involving 99Tc-nanocolloid injection (0.3–0.5 mCi) and isosulfan blue dye. Excised SLNs were evaluated intraoperatively by frozen section. Meanwhile, patients with positive SLNs proceeded to level I–II AD in the same surgical session. Decisions regarding axillary dissection were made intraoperatively in accordance with contemporary surgical oncology guidelines and multidisciplinary tumor board recommendations rather than individual surgeon discretion. Patients with residual axillary metastatic lymph nodes detected on imaging underwent axillary dissection.

### 2.2 Pathological and statistical analysis

Postoperative pathological findings were reviewed for all patients. Based on the axillary pathology results, patients were categorized into two groups:

**Group 1:** No residual axillary metastasis
**Group 2:** Residual axillary metastasis present

Axillary pathological complete response (pCR) was defined as ypN0 (absence of residual macrometastasis or micrometastasis). Isolated tumor cells [≤0.2 mm; ypN0(i+)] were considered node-negative. The two groups were compared for mean age, type of surgery, number of excised SLNs, and the presence of a complete pathological response in the primary tumor. Tumor characteristics, including invasive tumor size, estrogen receptor (ER) status, progesterone receptor (PR) status, HER2 status, histological grade, and Ki-67 index, were recorded and statistically analyzed between the two groups. The cut-off value for the Ki-67 index was accepted as 20%.

The diagnostic performance [sensitivity, specificity, positive predictive value [PPV], and negative predictive value [NPV]] of post-NACT imaging modalities in detecting axillary metastasis was calculated. The two groups were compared regarding mean age, tumor size, and metastatic lymph nodes using Student's *t*-test. Pearson's chi-square or Fisher's exact test was used to compare the groups for tumor characteristics, ER status, PR status, HER2 status, grade, Ki-67 index, lymphovascular invasion (LVI), perineural invasion (PNI), and operation type.

Statistically significant variables in univariate analyses (age, ER, PR, HER2, Ki-67, LVI, PNI) were included in a multivariate logistic regression model to evaluate the independent effects of these parameters on the axillary response rate after NACT using the Backward LR method. Variables with *p* < 0.10 in univariate analysis were entered into the multivariate model. Given the potential collinearity between ER, PR, HER2 status, and molecular subtype, these variables were not included simultaneously in the same regression model. Collinearity diagnostics were performed, and variance inflation factors (VIF) were evaluated. With 78 axillary pCR events, the number of covariates included in the final model was considered appropriate according to event-per-variable recommendations. Sensitivity, specificity, PPV, and NPV values were calculated for imaging modalities using pathological true positive and true negative outcomes. Statistical analyses were performed using SPSS version 25.0 (IBM Corp., Armonk, NY, USA). A *p*-value of < 0.05 was considered statistically significant. The study protocol was reviewed and approved by the institutional ethics committee of Katip Celebi University Medical Faculty (Approval No: 0181, 08.04.2025).

## 3 Results

The mean age of the patients included in the study was 52.0 years (range: 27–81 years). After completion of NACT, 98 patients (69.0%) achieved clinical and radiologic axillary downstaging and underwent SLNB. The remaining 44 patients underwent upfront level I–II axillary dissection due to persistent clinical and/or radiologic suspicion. Tumor characteristics and the types of surgeries performed are summarized in Table 1. Pathological examination of the surgical specimens revealed a pCR of the primary tumor in 64 patients (45.1%). Tumor stages of the remaining 78 patients after surgery were T1 in 61 patients (42.9%), T2 in 16 patients (11.2%), and T3 in 1 patient (0.9%). Of these 142 patients, 22.5% had multifocal or multicentric tumors based on pathological results.

**Table 1:** Clinicopathological characteristics of the patients.

Parameter	*N*: 142
Age	52.0 (27-81)
Tumor type	
IDC	126 (88.7%)
ILC	7 (4.9%)
Mixed	5 (3.5%)
Other	4 (2.9%)
ER	
Positive	103 (72.5%)
Negative	39 (27.5%)
PR	
Positive	85 (59.8%)
Negative	57 (41.2%)
HER2	
Positive	50 (35.2%)
Negative	92 (64.8%)
Ki-67	
<20%	34 (23.9%)
≥20%	108 (76.1%)
LVI	
Negative	109 (76.7%)
Positive	33 (23.3%)
PNI	
Negative	113 (79.5%)
Positive	29 (20.5%)
Breast operation	
SM	94 (66.1%)
TM	48 (33.9%)
Number of retrieved lymph nodes	
SLNB	4.2 (3–8)
AD	13.9 (8–26)
Mean metastatic lymph node number	4.2 (2–17)
Axilla operation	
SLNB	78 (54.9%)
SLNB + AD	20 (14.1%)
AD	44 (31.0%)

IDC, invasive ductal carcinoma; ILC, invasive lobular carcinoma; ER, estrogen receptor; PR, progesterone receptor; HER2, c-erbB2; LVI, lymphovascular invasion; PNI, perineural invasion; SM, segmental mastectomy; TM, total mastectomy; LN, lymph node; SLNB, sentinel lymph node biopsy; AD, axillary dissection.

Imaging modalities and clinical evaluation showed no evidence of axillary metastasis in 98 patients (69%), and these patients underwent SLNB, whereas the remaining 44 patients underwent level I–II AD. The median number of retrieved sentinel lymph nodes was 4. There was no significant difference in the number of retrieved SLNs between surgeons. Among the 98 patients, 20 were found to have metastatic lymph nodes and subsequently underwent level I–II AD in the same session. In 13 of these patients, the positive lymph nodes were identified as SLNs via blue dye or radiotracer. In the remaining 7 patients, the SLNs stained with blue dye or retaining radioactive material had no metastasis, and the lymph nodes detected by palpation during the operation were found to be metastatic. Thus, the false-negative rate for SLNB was 7.1% (7 out of 98 patients who underwent SLNB).

As a result of the final pathological examination, 78 patients (54.9%) had no residual axillary lymph node metastasis (i.e., Group 1), and 64 patients (45.1%) had residual axillary lymph node metastasis (i.e., Group 2). All patients in Group 2 underwent level I–II AD. The mean number of excised lymph nodes was 4.2 (range: 3–8) in Group 1 and 13.9 (range: 8–26) in Group 2. The mean number of positive lymph nodes across all patients was 4.2 (Table 1).

Of the 20 patients who underwent AD due to positive SLNB, 10 patients (50%) had no metastasis in non-sentinel nodes (non-SLNs). These 20 patients included 1 triple-negative, 1 HER2-rich, 12 luminal B, and 6 luminal A tumors. The initial staging was similar between the groups. The initial tumor and nodular stages are summarized in Table 2. T2 tumors were observed in 66.7% of patients in Group 1 and 60.9% of patients in Group 2, whereas N1 disease was seen in 76.9% of patients in Group 1 and 68.7% of patients in Group 2.

**Table 2:** The stages of the tumors before NACT based on the AJCC staging 8th edition.

	LN negative after NACT (78)	LN positive after NACT (64)
Tumor stage		
T1	21 (26.9%)	9 (14.1%)
T2	52 (66.7%)	39 (60.9%)
T3	5 (6.4%)	16 (25.0%)
Axillary LN stage		
N1	60 (76.9%)	44 (68.7%)
N2	18 (23.1%)	20 (31.3%)

NACT, neoadjuvant chemotherapy; AJCC, The American Joint Committee on Cancer; LN, lymph node.

Regarding tumor subtypes in the study group, 22 patients (15.4%) had luminal A, 85 (59.8%) had luminal B, 14 (9.8%) had HER2-rich, and 21 (14.7%) had triple-negative tumors. Post-NACT axillary response rates were 18.1%, 56.4%, 85.7%, and 71.4% in luminal A, luminal B, HER2-rich, and triple-negative tumors, respectively. Luminal A and B tumors demonstrated significantly lower response rates to NACT compared to other subtypes (*p* < 0.001).

Pathological analysis showed that ER-negative, PR-negative, and HER2-positive tumors were significantly more frequent in Group 1 than in Group 2 (*p* < 0.05). Moreover, lymphovascular invasion (LVI)-negative and perineural invasion (PNI)-negative tumors were significantly more common in Group 1 than in Group 2 (*p* < 0.001). Tumors with a Ki-67 index ≥20% showed significantly higher response rates (*p* < 0.001).

The results of the univariate analyses are summarized in Table 3. Pathological subtype, ER status, PR status, HER2 status, Ki-67 index, PNI status, and LVI status were significantly associated with axillary response to NACT. Variables with *p* < 0.10 in univariate analysis were entered into the multivariate logistic regression model. In multivariate analysis, PR negativity (*β* = 1.627, OR: 5.10, 95% CI: 2.021–12.819, *p* = 0.001), HER2 positivity (*β* = 1.529, OR: 4.60, 95% CI: 1.732–12.299, *p* = 0.002), absence of LVI (*β* = 1.944, OR: 6.98, 95% CI: 2.159–22.595, *p* = 0.001), and absence of PNI (*β* = 1.687, OR: 5.41, 95% CI: 1.528–19.120, *p* = 0.009) were independently associated with a higher likelihood of achieving axillary pCR (Table 4). The final model included four covariates with 78 axillary pCR events, corresponding to approximately 19 events per variable, exceeding the commonly recommended minimum threshold of 10 events per variable and supporting adequate model stability. Pathological subtype, ER status, and Ki-67 index were not independent predictors of residual axillary metastasis after adjustment.

**Table 3:** Statistical differences between groups (univariate analysis; complete-case analysis).

Parameter (*N*)	LN negative (78) (Group 1)	LN positive (64) (Group 2)	Missed data	*P* *
Age	52.26 ± 10.1	52.73 ± 11.8	0	0.9
Tumor type			0	0.6
IDC (126)	70 (55.6%)	56 (44.4%)		
ILC (7)	3 (43.9%)	4 (57.1%)		
Mixed (5)	2 (40%)	3 (60%)		
Other (4)	3 (75%)	1 (25%)		
Pathologic subtype				
Luminal A (22)	4 (18.1%)	18 (71.9%)		**0.001**
Luminal B (85)	48 (56.4%)	37 (43.6%)		
HER2-rich (14)	12 (85.7%)	2 (14.3%)		
TN (21)	15 (71.4%)	6 (18.6%)		
ER			0	**0**.**03**
Positive (103)	49 (47.6%)	54 (52.4%)		
Negative (39)	29 (74.4%)	10 (25.6%)		
PR			0	**0**.**0001**
Positive (85)	34 (40%)	51 (60.0%)		
Negative (57)	44 (77.2%)	13 (22.8%)		
HER2			0	**0**.**0001**
Positive (50)	41 (82.0%)	9 (18.0%)		
Negative (92)	37 (40.2%)	55 (59.8%)		
Grade			0	0.5
1 (24)	13 (54.1%)	11 (45.9%)		
2 (63)	35 (55.5%)	28 (44.5%)		
3 (55)	30 (54.5%)	25 (45.5%)		
Ki-67			0	**0**.**004**
<20 (34)	14 (41.2%)	20 (58.8%)		
≥20 (108)	64 (59.3%)	44 (40.7%)		
LVI			1	**0**.**0001**
Positive (32)	5 (15.6%)	27 (84.4%)		
Negative (109)	72 (66.1%)	37 (33.9%)		
PNI			1	**0**.**0001**
Positive (28)	4 (14.3%)	24 (85.7%)		
Negative (113)	73 (64.6%)	40 (35.4%)		

IDC, invasive ductal carcinoma; ILC, invasive lobular carcinoma; LN, lymph node; TN, triple negative; ER, estrogen receptor; PR, progesterone receptor; LVI, lymphovascular invasion; PNI, perineural invasion;

* *p* < 0.05 is significant.

The significant parameters were shown with bold writings.

**Table 4:** The statistical results of multivariate analysis.

	Beta	P*	Odds ratio	95% CI (Lower–Upper)	
				Lower	Upper
PR	1.627	0.001	5.1	2.021	12.819
HER2	1.529	0.002	4.6	1.732	12.299
LVI	1.944	0.001	6.984	2.159	22.595
PNI	1.687	0.009	5.405	1.528	19.120

PR, progesterone receptor; LVI, lymphovascular invasion; PNI, perineural invasion; *p < 0.05 is significant.

We also examined USG and PET-CT results in 142 patients, MG results in 32 patients, and MRI results in 60 patients after NACT. The diagnostic performance of imaging modalities in detecting axillary metastasis after NACT is summarized in Table 5. PET-CT and USG were the most valuable imaging modalities for predicting positive lymph nodes after NACT. The specificity values were 91.2% and 84.6% for PET-CT and USG, respectively. The performance of the imaging modalities in predicting positive lymph nodes was less than 70% according to PPV, except for PET-CT, which had a PPV of 75%. Although MRI and MG showed relatively high NPVs (87.5%), these findings should be interpreted cautiously due to the limited number of patients who underwent these imaging modalities. The NPVs for PET-CT and USG were 74.02% and 76.52%, respectively.

**Table 5:** The prediction values of imaging methods after NACT in axillary LNs.

Method (N)	Specificity	Sensitivity	PPV	NPV
USG(142)	84.62	52.63	65.22	76.52
MG (32)	87.5	**62.5**	62.5	**87**.**5**
MRI(60)	68.42	59.09	52.00	**87**.**5**
PET-CT (142)	**91**.**26**	45.00	**75**.**00**	74.02

N, number of patients who were performed imaging methods; NACT, neoadjuvant chemotherapy; LN, lymph node; USG, ultrasonography; MG, mammography; MRI, magnetic resonance imaging; PET-CT, 18F-fluorodeoxyglucose positron emission tomography-computed tomography; PPV, positive predictive value; NPV, negative predictive value.

The maximum values were shown by bold writings.

Regarding the results of imaging modalities in the 10 patients who had metastatic non-SLNs, PET-CT predicted positive non-SLNs in 7 of 10 (70%) patients, and USG in 8 of 10 (80%) patients. The results of other imaging modalities could not be obtained for these patients. The USG, PET-CT, MG, and MRI results of the remaining 10 patients who had no metastatic non-SLNs showed that USG predicted negativity in 8 of 10 patients (80%). PET-CT predicted negative non-SLNs in 6 of 10 patients (60%). Statistical analysis was not performed for non-SLNs data because of the limited numbers.

## 4 Discussion

SLNB is the commonly preferred axillary surgical approach in patients with BC showing a complete axillary response following NACT. However, SLNB after NACT may carry a certain risk of false-negative results. A false-negative SLN result is defined as the absence of cancer cells in the SLNs while metastasis is present in the remaining lymph nodes in the axillary region. Similar to our results, a meta-analysis showed a false-negative rate of 7.1% in patients who were clinically negative for axillary BC after NACT (13). Meanwhile, clinical studies have demonstrated that SLNB can be performed safely after NACT when dual tracers (i.e., radioisotope and blue dye) are used and at least three SLNs are retrieved.

According to the large SENTINA and ACOSOG Z1071 clinical trials, false-negative rates could be reduced to less than 10%—the generally acceptable threshold—when SLNB is performed using the dual-tracer technique and more than two SLNs are removed (8, 14). Of course, these studies had some limitations. The analysis of patients from whom more than two SLNs were extracted was unplanned in those studies (15). Furthermore, some patients were not pathologically proven to have axillary metastasis before NACT in the SENTINA study (14). However, these studies confirmed that the false-negative rate of SLNB after NACT falls below 10% when the dual-tracer method is utilized and three or more SLNs are removed. In our study, we performed SLNB using the dual-tracer method and extracted three or more SLNs. The median number of retrieved sentinel lymph nodes was 4 (range: 3–8), and ≥3 sentinel lymph nodes were removed in 100% of patients. Our false-negative rate was 7%, which is consistent with the literature.

To minimize the false-negative rate, the TAD technique has gained attention in recent years. Although literature shows that TAD yields highly promising outcomes, it has not yet become a standard or widely accessible procedure. The method has some limitations, including the requirement for additional procedures such as clip placement before NACT in suspicious lymph nodes and marking the clipped lymph node prior to surgery after NACT (16). The clipped lymph node must be identified pathologically during the operation, and in some patients, the clip could not be found. Additionally, in patients with clinical N2 disease according to pretreatment imaging, determining which and how many lymph nodes should be clipped remains a confusing issue. Despite these limitations, the false-negative rate for TAD is reported to be approximately 2%–3% (9, 17, 18).

In our study, none of the patients underwent TAD, primarily due to institutional resource limitations. Our clinic previously performed TAD in several patients; however, due to the inconsistent detection of clipped nodes after NACT, the practice was discontinued. In our study, SLNB was successfully performed in nearly all eligible patients. SLNs were accurately identified in 98 patients, and AD was subsequently performed in those with at least one positive SLN on frozen section analysis. As previously mentioned, our false-negative rate of 7% was acceptable for the SLNB procedure.

Deciding which patients can safely undergo SLNB after NACT is a critical problem for surgeons who need to reliably rule out residual metastatic lymph nodes. Imaging modalities play an important role in assessing the presence of residual axillary disease after NACT, and several studies have shown that USG, MRI, and PET-CT are effective in detecting nodal metastasis. Various studies have also demonstrated that the false-negative rate of SLNB after NACT decreases when supplemented by USG assessment (8, 19). In the ACOSOG Z1071 trial, the false-negative rate was reduced to 9.8% by incorporating USG-based axillary assessments (8). In the SN FNAC trial, investigators found similar results, achieving a low false-negative rate of 2.7% when using USG (19). Maeshima et al. (14) reported that the NPV of USG in predicting axillary lymph node status after NACT was 50.8%. They also noted that incorporating molecular subtype analysis improved the NPV of USG. Specifically, hormone receptor-negative and HER2-positive tumors showed marked improvement in NPV when evaluated with USG, reaching up to 85% in HER2-positive BC patients.

In our study, we did not evaluate the effect of molecular subtypes on the specificity, sensitivity, PPV, and NPV of imaging modalities. However, we did investigate the predictive values of four different imaging modalities. USG and PET-CT demonstrated high concordance with pathological findings for identifying metastatic lymph nodes following NACT in terms of specificity and sensitivity. The NPV based on USG in our study was 76.52%, which aligns with rates reported in the literature. Although MRI and MG yielded the highest NPV (87.5%), the number of patients who underwent these modalities after NACT was much lower compared to USG and PET-CT (60 patients underwent MRI and 30 underwent MG after NACT). The highest specificity among the methods was 91.2%, observed with PET-CT.

Another important issue in our patient population was the status of non-SLNs. After NACT, non-SLNs are found to be metastatic in 35%–55% of patients who have residual SLN metastasis (20, 21). Consequently, nearly 50% of these patients undergo an unnecessary AD (21). In our study, 50% of patients with positive SLNs had no additional metastasis in their non-SLNs; thus, these patients also received an unnecessary AD. Although statistical analysis could not be performed due to our limited sample size, the majority of patients without non-SLN metastasis had the luminal B subtype. As with residual metastatic SLNs, USG and PET-CT were capable of detecting the status of non-SLNs.

In recent years, omitting AD for axillary management in certain patients with positive SLNs following NACT has been proposed. Some retrospective studies suggest that AD may not always be necessary in patients with positive SLNs after NACT (22). However, definitive conclusions cannot be drawn without prospective randomized clinical trials. Studies have additionally shown that luminal A tumors tend to exhibit relatively low response rates to NACT, whereas HER2-enriched and triple-negative subtypes are associated with significantly higher rates of pCR. Clark et al. (23) reported that ER-positive, PR-positive, and HER2-negative tumors had low axillary response rates to NACT; they also observed that tumors with a Ki-67 index of less than 50% exhibited poor responses after NACT. Our results are consistent with these findings, demonstrating that triple-negative and HER2-positive tumors achieved high rates of complete axillary response.

In our study, multivariate analysis revealed that PR-negative and HER2-positive tumors had significantly higher complete axillary response rates following NACT. The response rate of primary tumors to NACT corresponded with the response rate of axillary lymph nodes in several studies, reaching approximately 70% (23, 24). We also found a strong correlation (77%) between the axillary response to NACT and the primary tumor response within the breast, which is consistent with the current literature. Concordance between breast and axillary pathological response was quantified using Cohen's kappa coefficient. Patients who achieved pathologically complete responses in the primary tumor were more likely to exhibit complete nodal response in the axilla as well.

This study has several limitations. First, its retrospective and single-center design may introduce selection bias, as treatment decisions and imaging assessments reflect institutional practice patterns. Second, information bias may have occurred due to reliance on medical records. Third, the absence of standardized imaging interpretation protocols may affect reproducibility. Another important limitation is the potential verification (work-up) bias in the evaluation of imaging performance. Surgical decision-making was partially guided by clinical and radiological findings, and not all patients underwent the same reference standard procedure initially. Patients with persistent radiological suspicion were more likely to undergo upfront axillary dissection, whereas those with radiologic complete response underwent SLNB. This approach may have influenced sensitivity and specificity estimates. Therefore, imaging performance results should be interpreted with caution.

## 5 Conclusion

SLNB performed using dual tracers and the removal of at least three SLNs can be safely applied for axillary staging following NACT. In patients with residual nodal disease detected by USG or PET-CT after NACT, SLNB should be performed with special caution. However, given their modest sensitivity, imaging modalities should be considered complementary tools rather than substitutes for pathological axillary staging. Particular attention should be given to patients with PR-positive and HER2-negative tumors, as they are less likely to achieve an axillary response. These findings likely reflect underlying tumor biology rather than isolated receptor status. In these subgroups, TAD may be considered an alternative method when institutional and patient conditions permit. Further prospective randomized trials are needed to define patient groups who may safely avoid axillary dissection after NACT

## Data Availability

Retrospective files were analyzed. Requests to access these datasets should be directed to the corresponding author.
